# Evaluation of Lateral-Flow Assay for Rapid Detection of Influenza Virus

**DOI:** 10.1155/2020/3969868

**Published:** 2020-09-08

**Authors:** Meng-Yi Han, Tian-Ao Xie, Jia-Xin Li, Hui-Jin Chen, Xiao-Hui Yang, Xu-Guang Guo

**Affiliations:** ^1^Department of Clinical Medicine, The Third Clinical School of Guangzhou Medical University, Guangzhou 511436, China; ^2^Department of Clinical Laboratory Medicine, The Third Affiliated Hospital of Guangzhou Medical University, Guangzhou 510150, China; ^3^Key Laboratory for Major Obstetric Diseases of Guangdong Province, The Third Affiliated Hospital of Guangzhou Medical University, Guangzhou 510150, China; ^4^Key Laboratory of Reproduction and Genetics of Guangdong Higher Education Institutes, The Third Affiliated Hospital of Guangzhou Medical University, Guangzhou 510150, China

## Abstract

**Background:**

Influenza virus mainly causes acute respiratory infections in humans. However, the diagnosis of influenza is not accurate based on clinical evidence, as the symptoms of flu are similar to other respiratory virus. The lateral-flow assay is a rapid method to detect influenza virus. But the effectiveness of the technique in detecting flu viruses is unclear. Hence, a meta-analysis would be performed to evaluate the accuracy of LFA in detecting influenza virus.

**Methods:**

Relevant literature was searched out in PubMed, Embase, Web of Science, and Cochrane Library databases with the keywords “lateral flow assay” and “flu virus”. By Meta-DiSc software, pooled sensitivity, pooled specificity, positive likelihood ratio (PLR), negative likelihood ratio (NLR), diagnostic odds ratio (DOR), summary receiver operating characteristic curve (SROC), and area under the curve (AUC) can be calculated.

**Results:**

This meta-analysis contains 13 studies and 24 data. The pooled sensitivity and specificity of the influenza virus detected by LFA were 0.84 (95% CI: 0.82-0.86) and 0.97 (95% CI: 0.97-0.98), respectively. The pooled values of PLR, NLR, DOR, and SROC were 32.68 (17.16-62.24), 0.17 (0.13-0.24), 334.07 (144.27-773.53), and 0.9877. No publication bias was found.

**Conclusions:**

LFA exhibited high sensitivity and specificity in diagnosing influenza virus. It is a valuable alternative method which can diagnose influenza virus quickly. However, more evidence is required to confirm whether LFA is comparable to traditional methods for detecting the virus.

## 1. Introduction

Influenza epidemic is a worldwide public health challenge that leads to substantial socioeconomic burden [1]. The World Health Organization (WHO) reported that every year across the globe, about 1 billion people catch the flu, among whom the severe cases reach three to five million, and 290,000 to 650,000 die from respiratory diseases caused by the flu [2].

Seasonal influenza is caused by influenza viruses, and the meteoric spread of this acute respiratory infectious disease poses a threat to people worldwide. Influenza virus belongs to the Orthomyxoviridae family, which has four subtypes: A, B, C, and D [3]. Influenza A and B viruses spreading cause seasonal epidemics [4]. Influenza C viruses were similar to influenza B viruses, which are known to cause relatively mild respiratory disease in humans [5]. Influenza D viruses with the potential for zoonotic and interspecies transmission were discovered last among the Orthomyxoviridae family; its mechanism is still in infancy and is unclear [6]. Therefore, this article mainly discusses A and B subtypes in influenza virus.

Clinical features of influenza patients are similar to those of patients infected with other respiratory viruses such as rhinovirus, respiratory syncytium virus, parainfluenza virus, and adenovirus. It makes the diagnosis of influenza based on clinical grounds alone potentially inaccurate [7]. Consequently, laboratory diagnostic tests are essential for the diagnosis of influenza.

Currently, for the laboratory diagnosis of influenza viruses, real-time reverse transcription-polymerase chain reaction (RT-PCR) and virus culture are examined as the gold standard [8]. However, virus culture results in periods of up to 10 days, reducing its utility for clinical management [9]. Although RT-PCR shows higher sensitivity than virus culture, and results are obtained within 4-6 hours after submitting the specimen. However, the highest cost of specialized equipment and expertise required cause RT-PCR to be rarely used [10, 11]. Meanwhile, the lateral flow assay (LFA) is a rapid diagnostic test which can detect and quantify analytes in biological fluids, and the results will be available within 5–30 min [12]. It is a simple, sensitive, and practical technique that can be used in the absence of laboratory infrastructure and without advanced biological protection equipment [13]. The basic principle is as follows: clinical samples were combined with labeled antibody; the antigen of antibody compounds in a solid substrate by the capillary action of lateral flow, in a visible signal of the reaction zone, and the excess of labeled antibody continue to migrate through the second antibody capture, which leads to the second colour belt; by measuring, comparing, and testing personnel's qualitative, semiquantitative, and quantitative determination of antigen under test, intuitive results can be obtained within a short time [14].

For a long time, LFA is a widely used technique in clinical practice, on account of its low costs of developing and ease of manufacture [15, 16]. In accordance with the diverse elements of recognition used, LFA can be classified into two categories. One review focuses on “lateral-flow immunoassay” (LFIA), which mentions that antibodies were attached into exclusive recognition function. The other, nucleic acid LFA (NALFA), applies to test PCR products [17].

However, inspectors are only satisfied with simple technical operations and lack the information to reasonably evaluate the clinical value and reliability of test results and the scientific nature of diagnostic test methods. Hence, this meta-analysis is going to assess the accuracy of LFA in detecting influenza virus to systematically review all relevant studies.

## 2. Materials and Methods

### 2.1. Study Design

We commenced this research from January 1, 2000, to November 1, 2019. The accuracy of LFA in the identification of influenza virus was systematically evaluated.

### 2.2. Search Strategy

Four investigators systematically sought the literature from PubMed, Embase, Web of Science, and the Cochrane Library from January 1, 2000, to November 1, 2019. Articles in those databases were filtered out with keywords by “LFA” OR “lateral-flow assays” OR “Lateral flow assay” OR “Lateral flow immunoassay” OR “Lateral flow immunochromatographic assay” AND “Influenza viruses[all synonyms]”. The articles we retrieved are imported into Endnote X9.3.3.

### 2.3. Adoption Criteria and Screening Guidelines

The adoption criteria were as follows: (1) samples of influenza virus were identified by the research method with LFA as the core technology or gold standard method; (2) sufficient data will be generated to form a 2 × 2 contingency table and will be applied to figure out sensitivity, specificity, diagnostic accuracy, and 95% CI, and English literature is required; (3) lateral flow assay is a core method for detecting influenza viruses; (4) the specimens involved in the literature are humans; and (5) specimen capacity was no fewer than forty.

The screening guidelines were as follows: (1) iterated articles; (2) the literature types other than article; (3) the samples to be studied are from species other than humans; and (4) the gold standard for testing virus was not be mentioned.

### 2.4. Data Extraction

According to adoption criteria and screening guidelines established beforehand, the literature was retrieved by four researchers independently. After the screening, two evaluators extracted data from the final 13 included literatures. The following data were extracted: author, year of publication, countries that conduct experiments, study design, and so on. If any discrepancy arises in the extracted data, it would be settled through negotiation or a third researcher. P-value <0.05 was considered as statistically significant at 95% confidence interval.

### 2.5. Quality Assessment

QUADAS-2 can be used to review diagnostic accuracy and served as the evaluation criterion for the quality assessment of the research. This evaluation tool included four aspects: patient selection, indicator testing, reference criteria, and process and time [18].

### 2.6. Data Analysis and Synthesis

Diagnostic OR (DOR), negative LR (NLR), positive LR (PLR), sensitivity, specificity, and corresponding 95% confidence intervals (CIs) were calculated by using Meta-DiSc analysis of the data in the 2 × 2 contingency table. The stochastic effect model was conducted to the description of the precision of LFA in diagnosing influenza viruses, and the results were drawn into the forest map. Stata software was used to draw a funnel plot and chiefly show the analysis of distribution deviation.

### 2.7. Subgroup Meta-Analyses

We performed a subgroup analysis of two possible sources of heterogeneity based on the characteristics of the included studies. Through the relevant literature, we speculated that the difference of the sample source and the gold standard would have a great impact on the detection. The literature was divided into four groups according to different viral sources: nasal swab, nasopharyngeal aspirates, nasopharyngeal swab, and oropharyngeal swab. The literature was divided into three groups according to different gold standards: virus culture, RT-PCR, and both virus culture and RT-PCR. We do the data analysis by using Meta-DiSc.

## 3. Results

### 3.1. Search Results

We obtained 204 articles after searching from the mentioned databases. 82 duplicate articles were eliminated. Of the remaining 122 articles, 88 relevant articles were excluded by screening the titles and abstracts according to the inclusion/exclusion criteria, two articles did animal experiment, nine articles were on basic research, two articles were not written in English, two articles had nothing to do with influenza virus, three articles lacked the use of the gold standard such as culture or RT-PCR, and 3 articles had a sample size that was insufficient. Finally, we included 13 articles in the full-text reviewing for meta-analysis [19–31]. An additional file shows these in more detail (Figure S1).

### 3.2. Characteristics of the Included Studies

From these 13 articles, we extracted 24 sets of data to complete 2 × 2 tables. In the process of data extraction, the researchers also recorded the feature information of each article, which is summarized in Table 1.

### 3.3. Meta-Analyzed Publications' QUADAS-2 Results

In order to better evaluate the level of articles included in the analysis, the four researchers used a unified assessment scale—the Quality Assessment of Diagnostic Accuracy Studies (QUADAS-2)—as a standard.  Table 2 shows consequences of the quality assessment of the 13 included literatures.

### 3.4. Publication Bias

**Figure 1:**
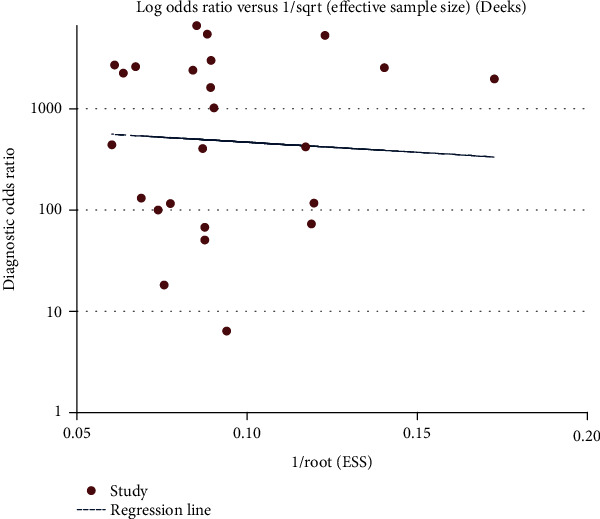
Deeks' funnel plot asymmetry test to assess publication bias in estimates of diagnostic odds ratio for LFA detection of influenza virus.

Deeks' funnel plot symmetry test was performed for the evaluation of publication bias in the included studies [32]. As shown in the funnel plots (Figure 1), most of the points are symmetrically distributed. Moreover, the *P* value of Deeks' test was 0.822 (*P* > 0.05), indicating that there was no publication bias.

### 3.5. The Analysis of Threshold Effect

**Figure 2:**
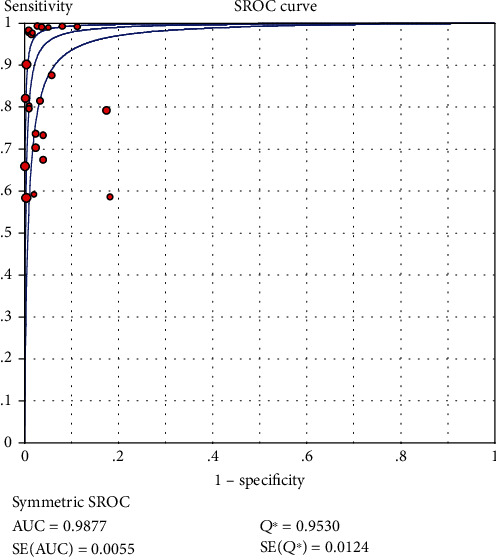
Summary receiver operating characteristic curves of influenza virus infections detected by LFA.

The Spearman correlation coefficient was 0.148 (<0.6) and the *P* value was 0.489 (*P* > 0.05) according to analyses. We also analyzed the SROC curve (Figure 2), which showed no “shoulder-arm” distribution. It was concluded that there were no threshold effects in the included studies.

### 3.6. SROC Curve

To assess the accuracy of LFA in diagnosing influenza viruses, we developed a SROC curve. As showed in Figure 2, AUC = 0.9877, and the Q index = 0.9530 (SE = 0.0124). Therefore, we can infer that LFA has a high accuracy in the diagnosis of influenza virus.

### 3.7. Merge Analysis Results

**Figure 3:**
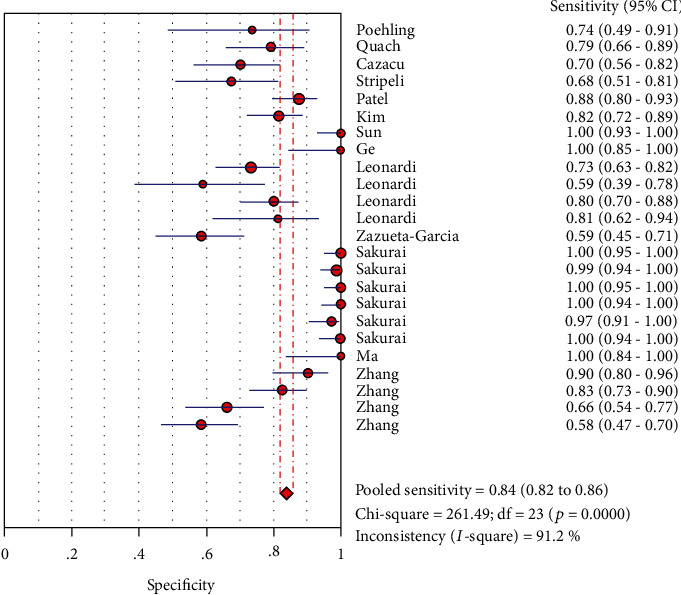
Forest plots for the pooled sensitivity of LFA.

**Figure 4:**
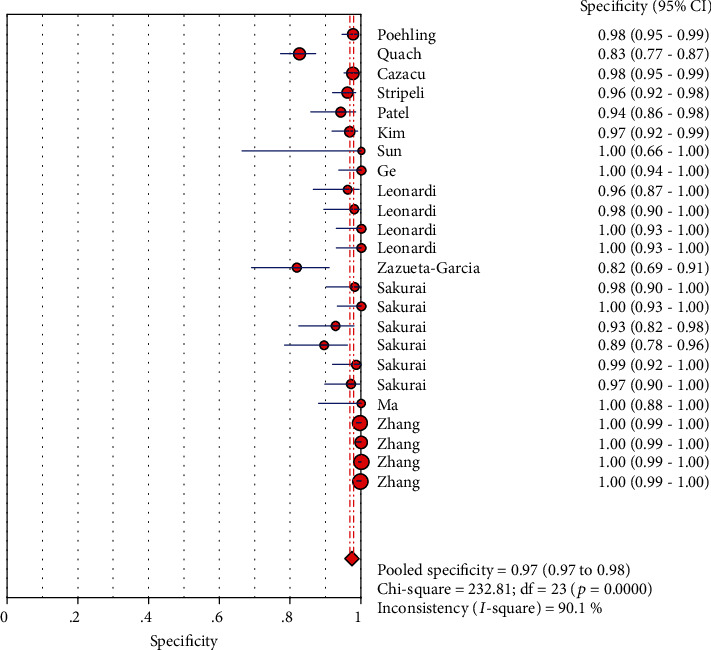
Forest plots for the pooled specificity of LFA.

**Figure 5:**
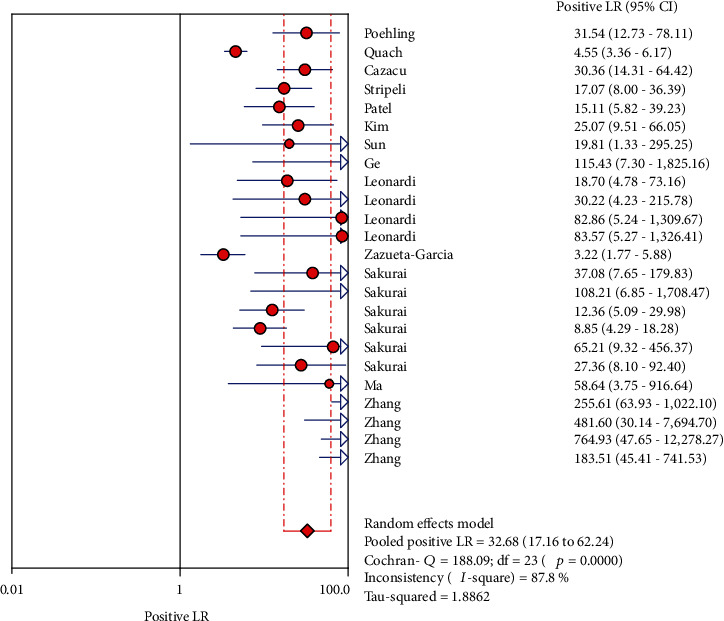
Forest plots for the pooled positive likelihood ratio of LFA.

**Figure 6:**
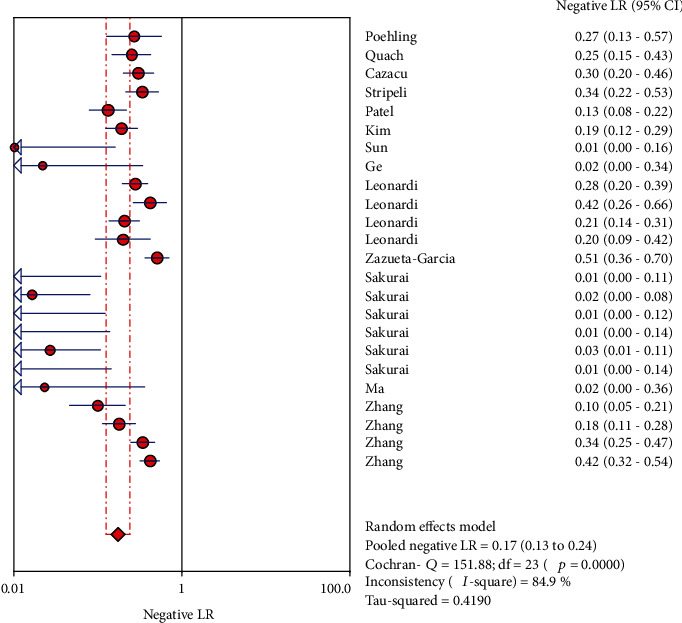
Forest plots for the pooled negative likelihood ratio of LFA.

**Figure 7:**
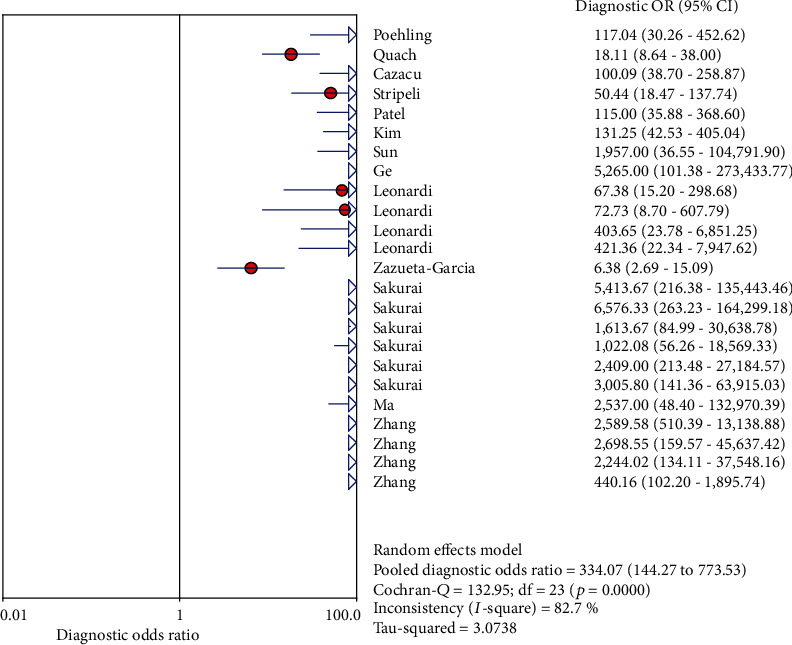
Forest plots for the pooled diagnostic odds ratio of LFA.

The analysis value was obtained by analyzing the 13 articles that were finally included. The results are as follows (the results are shown in Figures 3, 4, 5, 6, and 7): the sensitivity was 0.84 (95% CI (0.82, 0.86)), specificity was 0.97 (95% CI (0.97, 0.98)), positive likelihood ratio was 32.68 (95% CI (17.16, 62.24)), negative likelihood ratio was 0.17 (95% CI (0.13, 0.24)), and diagnostic odds ratio was 334.07 (95% CI (144.27, 773.53)).

### 3.8. Influenza Typing Analysis Results

**Figure 8:**
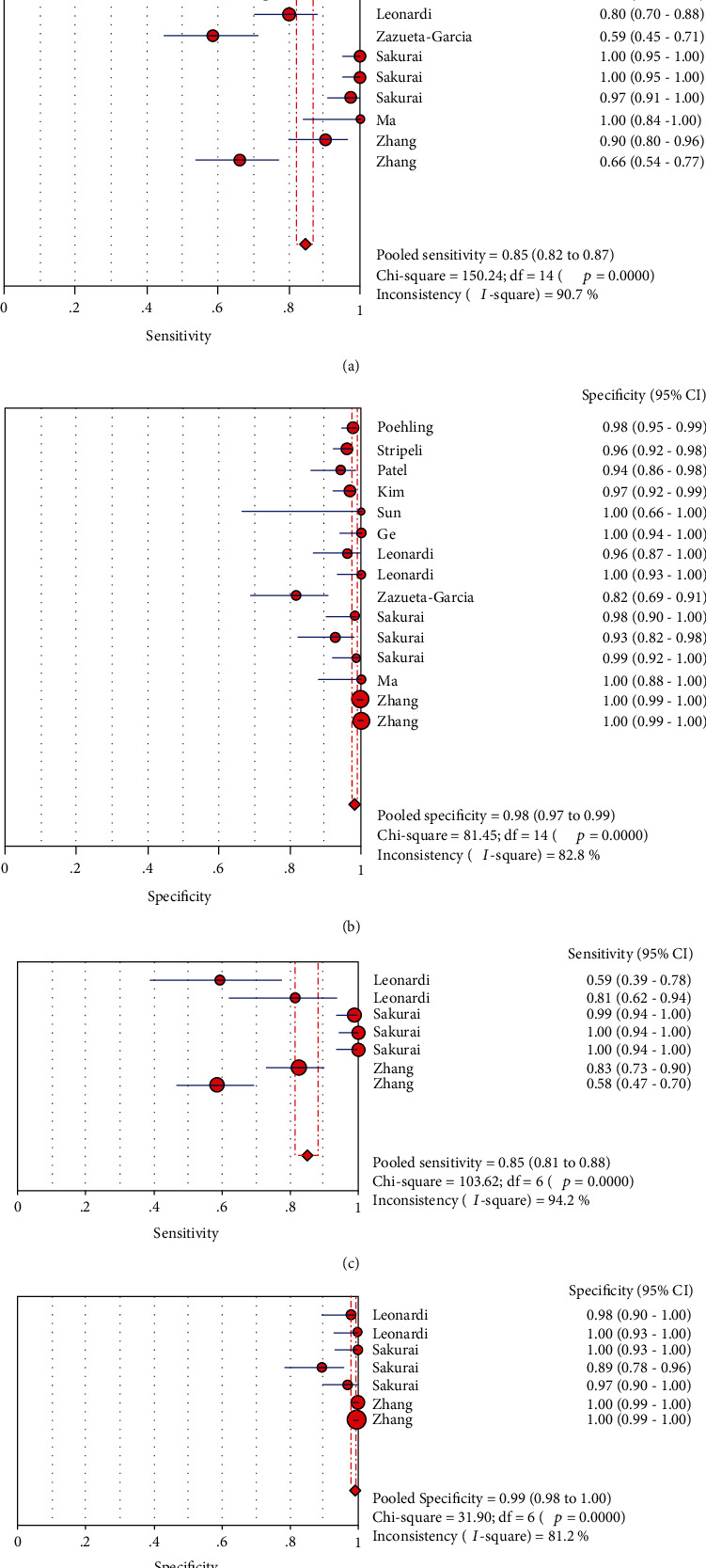
Forest plots for sensitivity and specificity of two types of influenza viruses: (a) forest plots for the pooled sensitivity of influenza virus A; (b) forest plots for the pooled specificity of influenza virus A; (c) forest plots for the pooled sensitivity of influenza virus B; (d) forest plots for the pooled specificity of influenza virus B.

In analyzing influenza virus A and influenza virus B separately, the results are shown in Figure 8: the sensitivity and specificity of influenza virus A were 0.85 (95% CI (0.82, 0.87)) and 0.98 (95% CI (0.97, 0.99)), respectively (Figures 8(a) and 8 (b)). The results of influenza virus B were 0.85 (95% CI (0.81, 0.88)) and 0.99 (95% CI (0.98, 1.00)), respectively (Figures 8(c) and 8(d)).

### 3.9. LFA Typing Analysis Results

**Figure 9:**
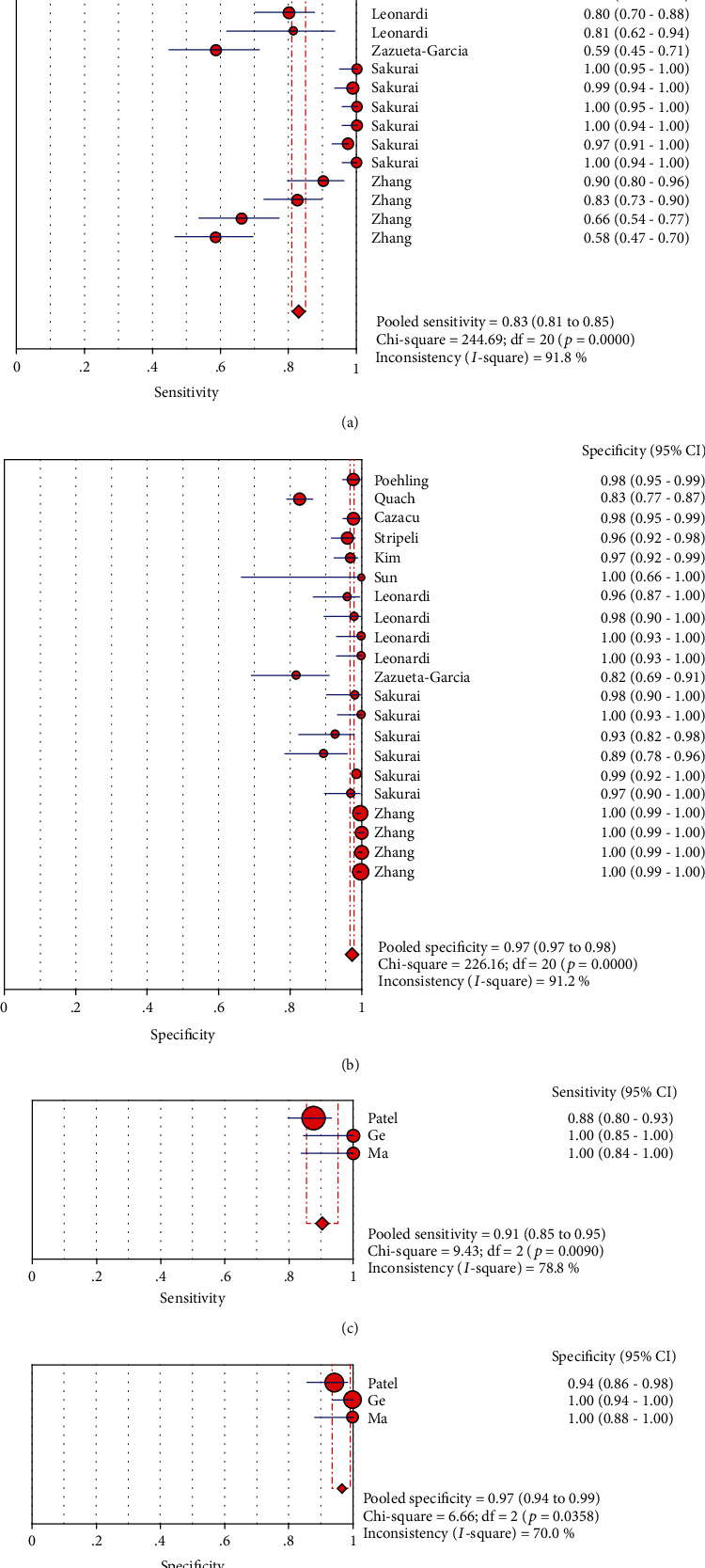
Forest plots for the sensitivity and the specificity of two types of LFA: (a) forest plots for the pooled sensitivity of LFIA; (b) forest plots for the pooled specificity of LFIA; (c) forest plots for the pooled sensitivity of NALFA; (d) forest plots for the pooled specificity of NALFA.

According to the different substances detected, LFA can be divided into LFIA and NALFA. The value was analyzed according to the classification. The results were as follows (Figure 9): sensitivity and specificity of LFIA were 0.83 (95% CI (0.81, 0.85)) and 0.97 (95% CI (0.97, 0.98)), respectively (Figures 9(a) and 9(b)). The results of NALFA were 0.91 (95% CI (0.85, 0.95)) and 0.97 (95% CI (0.94, 0.99)), respectively (Figures 9(c) and 9(d)).

### 3.10. Heterogeneity Analysis

A forest map is drawn using a random pattern. As is shown in Figure 7, diagnostic ratios for each study compared with the combined ratios are not along the same line. Moreover, a rough guide to quantitative indicators of heterogeneity by inconsistency index was interpreted as follows: 0–40%: low heterogeneity; 30–60%: moderate heterogeneity; 50–90%: significant heterogeneity; and 75–100%: considerable heterogeneity [33]. In our study, the following values can be obtained: Cochran − *Q* = 132.95, *P* < 0.001, and the inconsistency = 82.7% (inconsistency > 75%); this means that considerable heterogeneity existed in the nonthreshold effect. High heterogeneity was also detected across studies in other testings: sensitivity (*I*^2^ = 91.2%, *P* < 0.001), specificity (*I*^2^ = 90.1%, *P* < 0.001), PLR (*I*^2^ = 87.8%, *P* < 0.001), and NLR (*I*^2^ = 84.9%, *P* < 0.001).

### 3.11. Subgroup Meta-Analyses

The subgroup meta-analyses are summarized in Table 3.

For group A, test samples from different sources were used as subgroup analysis criteria, and the results were as follows:

*Nasal swab*: sensitivity was 0.88 (*P* < 0.001; *I*^2^ = 90.5%), and specificity was 0.97 (*P* = 0.3121; *I*^2^ = 15.8%).

*Nasopharyngeal aspirates*: sensitivity was 0.93 (*P* < 0.001; *I*^2^ = 90.6%), and specificity was 0.88 (*P* = 0.001; *I*^2^ = 91.7%).

*Nasopharyngeal*swab: sensitivity was 0.82 (*P* < 0.001; *I*^2^ = 83.0%), and specificity was 1.00 (*P* = 0.1105; *I*^2^ = 42.1%).

*Oropharyngeal swab*: sensitivity was 0.62 (*P* = 0.3374; *I*^2^ = 0.0%), and specificity was 1.00 (*P* = 0.1058; *I*^2^ = 61.8%).

For group B, different gold standard methods were used as criteria for subgroup analysis, and the results were as follows:

*Viral culture*: sensitivity was 0.75 (*P* = 0.2895; *I*^2^ = 10.9%), and specificity was 0.91 (*P* < 0.001; *I*^2^ = 97.5%).

*RT-PCR*: sensitivity was 0.85 (*P* < 0.001; *I*^2^ = 92.2%), and specificity was 0.99 (*P* < 0.001; *I*^2^ = 83.6%).

*Viral culture and RT-PCR*: sensitivity was 0.88 (P = 0.0035; *I*^2^ = 88.2%), and the pooled specificity was 0.98 (*P* = 0.1195; *I*^2^ = 58.7%).

## 4. Discussion

This study focused on evaluating the value of LFA in the diagnosis of influenza virus. After implementing certain screening criteria, we included a total of 24 data for analysis. The ultimate outcome of quality evaluation exhibited that the sensitivity and specificity of LFA in the identification of influenza virus were 0.84 and 0.97, respectively. The PLR, NLR, and DOR were 32.68, 0.17, and 334.07, respectively. The SROC AUC was 0.9877 (close to 1), indicating the high sensitivity and specificity of LFA in the identification of influenza viruses.

Subsequently, we used Stata software to make the Deeks funnel plot. When *P* > 0.05, it can be understood that no publication bias was found in the study [34]. The *P* value of the funnel plot is 0.822 (*P* > 0.05), so we took it to mean that no publication bias existed in our study. By drawing the SROC curve for each diagnostic approach, the heterogeneity caused by the threshold effect was probed into assessing if the points on the curve have a curve (shoulder-arm) pattern. The typical “shoulder-arm” pattern indicates a threshold effect [35]. However, when we analyzed the SROC curve for our study, we found that it had no “shoulder-arm” distribution. On the other hand, when the Spearman correlation coefficient is less than 0.6, the threshold effect is considered absent. In this study, the Spearman correlation coefficient was 0.148 (<0.6) and the *P* value was 0.489 (*P* > 0.05), which indicate that the included study had no threshold effect.

Furthermore, subgroup analyses were conducted to investigate heterogeneity in sensitivity and in specificity. The results of the subgroup analysis of the sample type indicated differences in the identification capabilities of sampling location. The results showed that the overall heterogeneity of nasopharynx aspirates was higher than that of the other three types. The *I*^2^ values of nasopharyngeal aspirates detected were 90.6%, respectively, suggesting high heterogeneity. In a comparison of the two sets of data with culture or RT-PCR as the gold standard, the results suggested that the specificity and sensitivity of both the RT-PCR and culture were higher than those of the group of only one gold standard. The reduction of sensitivity and specificity indicated that only culture or RT-PCR as a gold standard may lead to FP and FN results. Moreover, culture should not be regarded as a single gold standard. The analysis of culture in group B decreased significantly (*I*^2^ = 10.9%) indicating that reference criteria may not be a source of heterogeneity.

In addition to the above two sources of heterogeneity, we still consider some other possible sources of heterogeneity. For influenza virus samples, different laboratories have different processing methods such as different environments during specimen transportation and different concentrations of influenza virus in the collected samples, which will have a certain impact on the experimental results. Therefore, good sample handling can minimize the impact of environmental factors on virus activity. Generally speaking, the influenza virus should be stored in a virus preservation solution at a low temperature after collection until use, and repeated freezing and thawing should be avoided in this process [36]. The thermal stability of the virus will decrease with the increase of temperature. Repeated freezing and thawing and high temperature will reduce the stability of influenza virus RNA and accelerate the degradation of influenza virus RNA, thereby affecting the test results [37]. Thus, specimens should be submitted for inspection as soon as possible after collection. They should be submitted for inspection within 30 minutes at room temperature and within 2 to 4 hours at 4°C. Specimens that are too late to be processed should not be stored at 4°C for more than 48 hours. If possible, delivery is delayed for 24 hours, and specimens should be stored at <-70°C [38]. Due to the different ages of the tested patients, the sensitivity of LFA is also slightly different. Some articles speculate that influenza viruses are easier to isolate and detect in older patients [21]. Regarding the technology itself, LFA mainly relies on immune recognition, nucleic acid hybridization, and antibody labeling technology, in which the label is one of the key factors that affect its sensitivity [12]. The literature included in this meta-analysis shows that there are many types of markers used in different laboratories, such as biotin, luciferin, colloidal gold, superparamagnetic nanoparticles, and horseradish peroxidase, that affect the positive rate of LFA test results.

There is no doubt that RT-PCR or cell culture has higher accuracy of detecting influenza virus [11]. The accuracy of RT-PCR to detect influenza virus is slightly higher than that of culture [39]. The sensitivity and specificity of LFA in general show 0.85 and 0.99 compared to RT-PCR in our research. However, compared with cell culture, the sensitivity and specificity of LFA are 0.75 and 0.91. We found that using RT-PCR as the gold standard improves the accuracy of LFA detection. This may be the reason why RT-PCR has become more common as the gold standard for influenza virus detection in recent years.

Compared with using RT-PCR or culture, the sensitivity of using LFA to detect influenza virus of four sample types is nasopharyngeal aspirate (93%) > nasal swab (88%) > nasopharyngeal swab (82%) > oropharyngeal swab (62%), and specificity, nasopharyngeal swab = oropharyngeal swab (100%) > nasal swab (97%) > nasopharyngeal aspiration (88%), relatively speaking. Nasopharyngeal aspirates have a higher positive detection rate, and nasopharyngeal aspirate is more suitable for detecting respiratory viruses than throat swab [36]. Therefore, nasopharyngeal aspirate may be more suitable for LFA detection.

We analyzed the results of the subgroup analysis of the 13 included articles, from which we found that nasopharyngeal aspirates had the highest sensitivity in the four categories of appeal samples. This may be due to the fact that nasopharyngeal aspirates have a higher viral load than pharyngeal swabs in respiratory infection virus specimens, which makes nasopharyngeal aspirates easier to detect, and other researchers have shown in experiments that nasopharyngeal aspirates have a higher sensitivity than pharyngeal swabs [36, 40]. In addition, both nasopharyngeal aspirates and pharyngeal swabs belong to upper respiratory tract specimens. Compared with upper respiratory tract specimens, airway aspirates, alveolar lavage fluid, and other lower respiratory tract specimens have better sensitivity, but they cannot be widely used due to the difficulties in the collection process [41]. At the same time, there are more literature showing that although nasopharyngeal aspirates are more sensitive than pharyngeal swabs, the improved detection sensitivity of nasopharyngeal swabs is no less than that of nasopharyngeal swabs. Moreover, pharyngeal swabs are more popular than nasopharyngeal swabs due to their convenience and speed of collection [42]. Therefore, nasopharyngeal aspirates are superior to pharyngeal swabs in terms of sensitivity alone, but the practicality of pharyngeal swabs is greater when combined.

Virus type also had an effect on the accuracy of LFA. In the 4 articles included, it is mentioned that the sensitivity of LFA to detect influenza A virus is more effectively than that of influenza B virus. And the sample specimens of them were generally nasopharyngeal swabs and nasopharyngeal aspirates [21, 25, 27, 31]. However, in our study, the sensitivity of LFA to detect influenza viruses A and B was not significantly different. Further analysis found that in one of the included literatures, LFA was more sensitive in detecting influenza virus B than influenza virus A in nasal swabs [29]. Therefore, we infer that collecting nasal swab samples may enhance the sensitivity of detecting influenza virus B.

Furthermore, we analyzed the results of two different types of LFA tests. It was found that the sensitivity of NALFA to detect influenza virus is higher than that of LFIA, and there is no obvious difference between the specificities. The core of NALFA is nucleic acid hybridization, which captures and detects nucleic acid amplification products similar to lateral-flow immunoassays [43]. The combination of NALFA and amplification sample preparation technology, such as the loop-mediated isothermal amplification (LAMP) method, recombinase polymerase amplification (RPA), and rapid amplification/hybridization reaction, might make up for the lack of qualitative or semiquantitative LFA, improving its accuracy in rapid detection. In addition, the sensitivity of NALFA depends to a certain extent on the virus concentration of respiratory samples, and a higher virus concentration can produce a rapid positive result [23]. The virus concentration in respiratory samples is related not only to the type of virus and the organs or systems involved but also to host factors such as the patient's age and immune function status [44]. And the amount of virus secretion in the body varies with the course of the patient's disease and the location of the sample [45]. Therefore, the variability of sample sources will have a certain impact on the sensitivity of NALFA and LFIA test results.

In the literature we have included, LFIA is divided into the classic LFIA method and the improved LFIA method. Most of the principles of the classic LFIA methods and the improved LFIA methods are antigen-antibody reactions. The main difference lies in the different labels, which have a certain impact on the sensitivity of detection results. However, we have not yet retrieved the literature to compare and evaluate the performance of the classic LFIA method and the improved LFIA method, so we are unable to determine whether the improved LFIA method is more sensitive.

Our study has the following limitations: first, LFA cannot distinguish between influenza viruses. In addition, it is not clear that the impact of the accuracy of LFA technology in diagnosing influenza virus whether has effect on the age of patients. Because we have not contacted the authors, the age of the sampled patients in many of the included literature is not clear. Therefore, children and adults cannot be clearly separated. Although the overall sensitivity of LFA detection is very high, the results are not robust. Exactly how to improve the stability sensitivity of the detection results in various situations remains to be studied.

In summary, LFA is a fast, affordable, accurate, and thus promising method for detecting influenza viruses and is expected to have greater achievements for the diagnosis of influenza viruses than the current gold standard method.

## 5. Conclusion

In conclusion, our study demonstrates that LFA has high sensitivity and specificity in the diagnosis of influenza virus. More efforts should be made to define the accuracy of this promising test for diagnosing influenza virus in the future.

## Figures and Tables

**Table 1:** Characteristics of studies included in the meta-analysis.

Author	Year	Country	Study design	Reference standard method	Test technology	Rapid influenza tests	Age stage	Sample type	Sample source	Type of influenza virus	**The number** **of samples**	TP	FP	FN	TN
Poehling et al.	2002	The United States	Prospective	Culture+PCR	LFIA	QuickVue	Children (0 to 19 years old)	Nasal swab	Hospital	Influenza A (H3N2)	233	14	5	5	209
Quach et al.	2002	Canada	Prospective	Culture	LFIA	QuickVue	Children	Nasopharyngeal aspirates	Hospital	Influenza A and B virus	300	42	43	11	204
Cazacu et al.	2003	The United States	Prospective	Culture	LFIA	QuickVue	Children (3 months to 28 years)	Nasal wash specimens	Hospital	Influenza A and B virus	356	38	7	16	295
Stripeli et al.	2010	Greece	Prospective	RT-PCR	LFIA	QuickVue	Children (6 months to 14 years old)	Nasal swab	Pediatrician	Influenza A virus (H3N2)	217	27	7	13	170
Patel et al.	2011	Germany	Prospective	RT-PCR	NALFA	The rapidSTRIPE assay	Unclear	Nasal swab	Patient centers	Influenza A (H1N1)	174	92	4	13	65
Kim et al.	2012	Korea	Prospective	RT-PCR	LFIA	RDT kit	Unclear	Nasal swab	Hospital	Influenza A (H1N1)	215	75	4	17	119
Sun et al.	2013	China	Retrospective	RT-PCR	LFIA	IC-SED	Unclear	Nasopharyngeal swab	Hospital	Influenza A (H1N1)	60	51	0	0	9
Ge et al.	2013	China	Prospective	Culture+RT-PCR	NALFA	RT-LAMP-LFD	Unclear	65 pharyngeal swabs, 7 sputa, and 8 tracheal aspirates	Unclear	Influenza A (H7N9)	80	22	0	0	58
Leonardi et al.	2013	The United States	Prospective	RT-PCR	LFIA	QuickVue	Children 12 and under vs. adults	Nasopharyngeal swab	Unclear	Influenza A virus	141	66	2	24	49
Leonardi et al.	2013	The United States	Prospective	RT-PCR	LFIA	QuickVue	Children 12 and under vs. adults	Nasopharyngeal swab	Unclear	Influenza B virus	78	16	1	11	50
Leonardi et al.	2013	The United States	Prospective	RT-PCR	LFIA	Sofia	Children 12 and under vs. adults	Nasopharyngeal swab	Unclear	Influenza A virus	141	72	0	18	51
Leonardi et al.	2013	The United States	Prospective	RT-PCR	LFIA	Sofia	Children 12 and under vs. adults	Nasopharyngeal swab	Unclear	Influenza B virus	78	22	0	5	51
Zazueta-Garcia et al.	2014	Mexico	Retrospective	RT-PCR	LFIA	The Xpect Flu A&B Kit	Children	Nasopharyngeal washes	Hospital	Influenza A (H1N1)	113	34	10	24	45
Sakurai et al.	2015	Japan	Prospective	RT-PCR	LFIA	LFIC-AB	0 to 77 years old	Nasal swab	Unclear	Influenza A	129	74	1	0	54
Sakurai et al.	2015	Japan	Prospective	RT-PCR	LFIA	LFIC-AB	0 to 77 years old	Nasal swab	Unclear	Influenza B	145	90	0	1	54
Sakurai et al.	2015	Japan	Prospective	RT-PCR	LFIA	LFIC-AB	2 to 77 years old	Self-blow nasal discharge specimens	Unclear	Influenza A	125	70	4	0	51
Sakurai et al.	2015	Japan	Prospective	RT-PCR	LFIA	LFIC-AB	2 to 77 years old	Self-blow nasal discharge specimens	Unclear	Influenza B	121	64	6	0	51
Sakurai et al.	2015	Japan	Prospective	RT-PCR	LFIA	LFIC-AB	0 to 45 years old	Nasopharyngeal aspirates	Unclear	Influenza A	142	73	1	2	66
Sakurai et al.	2015	Japan	Prospective	RT-PCR	LFIA	LFIC-AB	0 to 45 years old	Nasopharyngeal aspirates	Unclear	Influenza B	124	56	2	0	66
Ma et al.	2018	China	Prospective	RT-PCR	NALFA	LFD-RPA	Unclear	Swabs and serum	Center for Disease Control and Prevention (CDC)	Influenza A (H7N9)	50	21	0	0	29
Zhang et al.	2019	China	Prospective	RT-PCR	LFIA	HRP-LFIA	Unclear	Nasopharyngeal swab	Centers for Disease Control and Prevention (CDC)	Influenza A	628	55	2	6	565
Zhang et al.	2019	China	Prospective	RT-PCR	LFIA	HRP-LFIA	Unclear	Nasopharyngeal swab	Centers for Disease Control and Prevention (CDC)	Influenza B	390	71	0	15	292
Zhang et al.	2019	China	Prospective	RT-PCR	LFIA	HRP-LFIA	Unclear	Oropharyngeal swab	Centers for Disease Control and Prevention (CDC)	Influenza A	647	45	0	23	579
Zhang et al.	2019	China	Prospective	RT-PCR	LFIA	HRP-LFIA	Unclear	Oropharyngeal swab	Centers for Disease Control and Prevention (CDC)	Influenza B	705	45	2	32	626

**Table 2:** The quality evaluation results for each study included in the meta-analysis.

Author	Year	QUADAS-2										
		1	2	3	4	5	6	7	8	9	10	11
Poehling et al.	2002	Y	Y	Y	UC	UC	Y	UC	Y	N	Y	N
Quach et al.	2002	Y	Y	Y	Y	UC	Y	Y	Y	Y	Y	N
Cazacu et al.	2003	Y	Y	Y	UC	UC	Y	UC	Y	Y	Y	Y
Stripeli et al.	2010	Y	Y	Y	Y	UC	Y	UC	Y	Y	Y	N
Patel et al.	2011	Y	UC	Y	UC	Y	Y	UC	Y	Y	Y	Y
Kim et al.	2012	Y	UC	Y	N	UC	Y	Y	Y	Y	Y	Y
Sun et al.	2013	Y	N	Y	N	UC	Y	Y	Y	Y	Y	Y
Ge et al.	2013	Y	Y	Y	Y	UC	Y	Y	Y	Y	Y	Y
Leonardi et al.	2013	Y	N	Y	N	UC	Y	Y	Y	Y	Y	Y
Zazueta-Garcia et al.	2014	Y	Y	Y	N	UC	Y	Y	Y	Y	Y	Y
Sakurai et al.	2015	Y	Y	Y	UC	UC	Y	UC	Y	Y	Y	Y
Ma et al.	2018	Y	N	Y	N	UC	Y	Y	Y	Y	Y	Y
Zhang et al.	2019	Y	Y	Y	N	UC	Y	Y	Y	Y	Y	Y

**Table 3:** Subgroup analysis results.

Subgroup analysis	Number of studies	Sensitivity (95% CI)	*I* ^2^	Specificity (95% CI)	*I* ^2^
Group A					
Nasal swab	6	0.88 (0.85-0.91)	90.5%	0.97 (0.95-0.98)	15.8%
Nasopharyngeal aspirates	3	0.93 (0.88-0.96)	90.6%	0.88 (0.84-0.91)	91.7%
Nasopharyngeal swab	7	0.82 (0.78-0.85)	83.0%	1.00 (0.99-1.00)	42.1%
Oropharyngeal swab	2	0.62 (0.54-0.70)	0.0%	1.00 (0.99-1.00)	61.8%

Group B					
Culture	2	0.75 (0.65-0.83)	10.9%	0.91 (0.88-0.93)	97.5%
PCR	20	0.85 (0.83-0.86)	92.2%	0.99 (0.98-0.99)	83.6%
Culture+PCR	2	0.88 (0.74-0.96)	88.2%	0.98 (0.96-0.99)	58.7%

CI: confidence interval.

## Data Availability

All data generated or analyzed during this study are included in this published article and its supplementary information files.

## Supplementary Materials

Supplementary Materials Additional file 1. Figure S1: flow chart of the literature review.
