# Supplementary figures and images for: Evaluation of Lateral-Flow Assay for Rapid Detection of Influenza Virus
Source: Biomed Res Int. 2020 Sep 8;2020:3969868. doi: 10.1155/2020/3969868 (PMC7495160; doi:10.1155/2020/3969868)

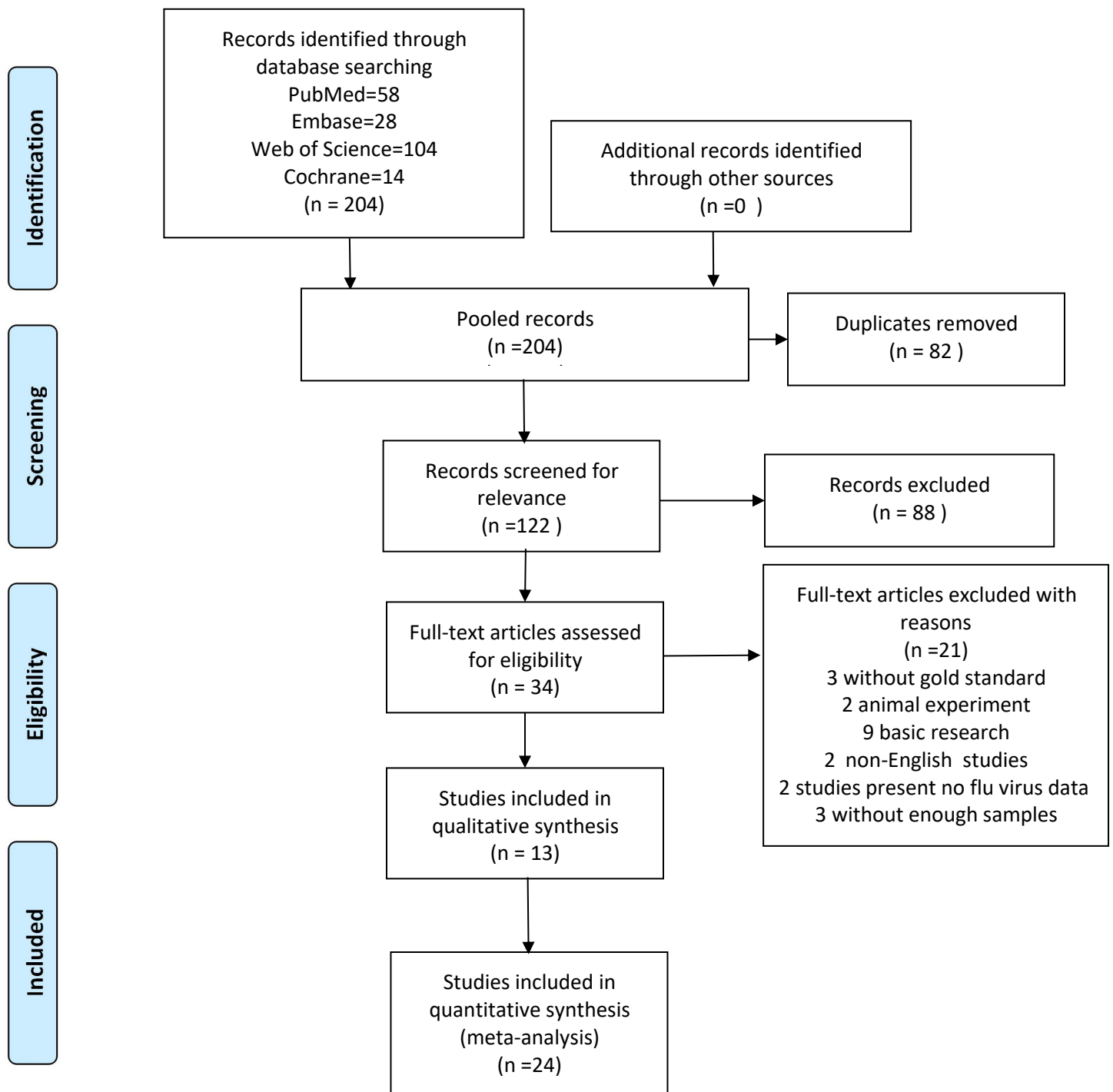

Supplement: Supplementary Materials — Additional file 1. Figure S1: flow chart of the literature review. [file 3969868.f1.pdf]
